# Prevalence of admission plasma glucose in 'diabetes' or 'at risk' ranges in hospital emergencies with no prior diagnosis of diabetes by gender, age and ethnicity

**DOI:** 10.1002/edm2.140

**Published:** 2020-05-15

**Authors:** Sandip Ghosh, Susan E. Manley, Peter G. Nightingale, John A. Williams, Radhika Susarla, Irene Alonso‐Perez, Irene M. Stratton, Georgios V. Gkoutos, Jonathan Webber, Stephen D. Luzio, Wasim Hanif, Graham A. Roberts

**Affiliations:** ^1^ Diabetes Translational Research Group Diabetes Centre Nuffield House Queen Elizabeth Hospital Birmingham Birmingham UK; ^2^ Institute of Translational Medicine Heritage Building (Queen Elizabeth Hospital) Birmingham UK; ^3^ Institute of Metabolism and Systems Research College of Medical and Dental Sciences University of Birmingham Birmingham UK; ^4^ Institute of Cancer and Genomic Sciences College of Medical and Dental Sciences University of Birmingham Birmingham UK; ^5^ Mammalian Genetics Unit Medical Research Council Harwell Institute Oxfordshire UK; ^6^ Health Informatics Department Queen Elizabeth Hospital Birmingham Birmingham UK; ^7^ Gloucestershire Retinal Research Group Cheltenham General Hospital Cheltenham UK; ^8^ MRC Health Data Research UK (Central Office) Gibbs Building London UK; ^9^ NIHR Biomedical Research Centre Birmingham UK; ^10^ Diabetes Research Unit (Cymru) Grove Building Swansea University Swansea UK; ^11^ HRB‐Clinical Research Facility – Cork Mercy University Hospital Cork Ireland

**Keywords:** emergency admissions, hyperglycaemia, undiagnosed diabetes

## Abstract

**Aims:**

To establish the prevalence of admission plasma glucose in 'diabetes' and 'at risk' ranges in emergency hospital admissions with no prior diagnosis of diabetes; characteristics of people with hyperglycaemia; and factors influencing glucose measurement.

**Methods:**

Electronic patient records for 113 097 hospital admissions over 1 year from 2014 to 2015 included 43 201 emergencies with glucose available for 31 927 (74%) admissions, comprising 22 045 people. Data are presented for 18 965 people with no prior diagnosis of diabetes and glucose available on first attendance.

**Results:**

Three quarters (14 214) were White Europeans aged 62 (43‐78) years, median (IQ range); 12% (2241) South Asians 46 (32‐64) years; 9% (1726) Unknown/Other ethnicities 43 (29‐61) years; and 4% (784) Afro‐Caribbeans 49 (33‐63) years, *P* < .001. Overall, 5% (1003) had glucose in the 'diabetes' range (≥11.1 mmol/L) higher at 8% (175) for South Asians; 16% (3042) were ‘at risk’ (7.8‐11.0 mmol/L), that is 17% (2379) White Europeans, 15% (338) South Asians, 14% (236) Unknown/Others and 11% (89) Afro‐Caribbeans, *P* < .001. The prevalence for South Asians aged <30 years was 2.1% and 5.2%, respectively, 2.6% and 8.6% for Afro‐Caribbeans <30 years, and 2.0% and 8.4% for White Europeans <40 years. Glucose increased with age and was more often in the 'diabetes' range for South Asians than White Europeans with South Asian men particularly affected. One third of all emergency admissions were for <24 hours with 58% of these having glucose measured compared to 82% with duration >24 hours.

**Conclusions:**

Hyperglycaemia was evident in 21% of adults admitted as an emergency; various aspects related to follow‐up and initial testing, age and ethnicity need to be considered by professional bodies addressing undiagnosed diabetes in hospital admissions.

## Graphical abstract

Glucose needs to be measured in all adults admitted as an emergency not just those over 40/30 years old, as younger people particularly South Asians and Afro‐Caribbeans are at risk of being admitted with undiagnosed diabetes. Overall, 5% of emergency admissions had glucose in the ‘diabetes’ range, 8% if South Asian and Afro‐Caribbean, with 16% of people in the ‘at risk’ range. One third of all emergency admissions were for <24 hours with 58% of these having glucose measured compared to 82% with duration >24 hours.

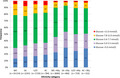

## 1 INTRODUCTION

The current diabetes pandemic threatens both the health and economy of nations.^1^ The prevalence is increasing year by year adding markedly to the cost of health care funded by governments or private healthcare organizations. Diabetes currently consumes over 10% of the UK National Health Service (NHS) budget.^2^ The prevalence of type 2 diabetes and its complications vary by ethnicity with type 2 diabetes more prevalent in people of South Asian descent (six times) and in Africans and Afro‐Caribbeans (three times) than White Europeans.^3^, ^4^

Symptoms of diabetes are not always evident until people consult a family doctor or are admitted to hospital.^5^ Undiagnosed diabetes results in an eightfold increase in mortality for hospital admissions compared to those with normal glucose.^6^ Admission glucose is strongly associated with mortality in acutely ill medical patients^7^ with hyper/hypoglycaemia independent predictors of in‐hospital mortality in patients not previously diagnosed with diabetes.^8^, ^9^

The American Diabetes Association guidance published in 2020 recommends measuring HbA1c in patients admitted with hyperglycaemia, defined as glucose >7.8 mmol/L.^6^ Although HbA1c is used for diagnosis in the community in the UK following WHO guidance in 2011,^10^ it is not currently requested routinely on hospital admission for this purpose. Data from an Irish hospital indicates that HbA1c could be used for follow‐up testing^11^ although it is not suitable for people with some haemoglobinopathies or altered red blood cell turnover.^10^

This clinical audit reports on admission plasma glucose in emergency admissions with no prior diabetes coding using laboratory and demographic data from hospital electronic patient records over 1 year. It aims to investigate people with glucose recorded on admission and no prior diabetes diagnosis, to categorize plasma glucose by ‘at risk’ and ‘diabetes’ ranges, and describe relationships to age, gender and ethnicity.

## 2 METHODS

### 2.1 Design

The clinical audit at Queen Elizabeth Hospital Birmingham was approved according to clinical governance (*CARMS‐12031*), University Hospitals Birmingham NHS Foundation Trust. This hospital, located in the West Midlands of England with a culturally diverse catchment, is a major trauma centre for adults comprising >1200 beds with 100 critical care beds.

### 2.2 Admissions

**FIGURE 1:**
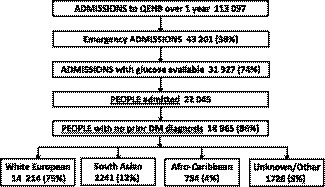
Flow chart for emergency admissions to a UK hospital located in a multi‐ethnic region over 1 y: 2014‐2015

There were 113 097 admissions between April 2014 and March 2015 with 38% (43 201) emergencies, others elective admissions or day care patients (Figure 1). Self‐reported ethnicity was coded as White European/Caucasian (British, Irish or any other white background); South Asian (Indian, Pakistani, Bangladeshi or any other Asian background); Afro‐Caribbean (Caribbean, African or any other black background); other groups (Chinese, any other ethnic groups not described above, and mixed ethnic groups) and unknown (those not specified and missing). Diabetes status was assigned on admission from ICD 10 coding (International Classification of Diseases, Tenth Revision). People with multiple admissions were identified, and their first admission designated as the index admission.

### 2.3 Study data

Data were obtained from the electronic patient record system PICS (patient information and communication system) with the initial plasma glucose result measured by point‐of‐care testing or in the laboratory.

### 2.4 Measurements

Blood glucose was measured at point‐of‐care on the wards in random capillary blood on glucose meters or arterial/venous whole blood samples on gas machines with results reported as plasma; Roche Cobas Inform II glucose meters (CV 5%) and Roche Cobas b221 blood gas machines (CV 4%). Blood was collected into fluoride oxalate vacutainers for measurement of plasma glucose in the central hospital laboratory using Roche Cobas 8000 analysers (CV 2%). Internal quality control and external quality assurance were overseen by the hospital blood sciences laboratory. The performance of the point‐of‐care equipment was compared with the laboratory analysers and found to be acceptable.

### 2.5 Statistical analysis

Anonymized clinical audit data were downloaded by Health Informatics, stored and analysed by a hospital statistician and data visualization analyst using Microsoft Excel, IBM SPSS Statistics for Windows version 22.0 (IBM Corp.) and R version 3.4.0 including ggplot2 and vcd packages.^12^, ^13^, ^14^

Variables are presented in Tables 1 and 2 as median and interquartile range, or count/percentage. Mann‐Whitney or Fisher's exact tests were used to compare groups. The association between the prevalence of prior diabetes diagnosis coding and number of admissions was assessed using Kendall's tau‐*b* statistic.

**TABLE 1:** Admission plasma glucose in emergency admissions over 1 y from 2014 to 2015

	All emergency admissions^a^	Admissions with glucose available^b^	People with glucose available	People with glucose available and no prior diabetes coding^b^, ^c^
n	43 201	31 927 (74%)	22 045	18 965 (86%)
Age^d^, y	60 (41‐77)	62 (43‐77)^e^, ***	60 (41‐76)	58 (38‐76)^e^, ***
Female	21 658 (50%)	15 947 (74%)	10 936	9539 (87%)***
Male	21 233 (50%)	15 744 (74%)	10 982	9325 (85%)
White European (WE)	32 581 (75%)	23 942 (73%)	16 271	14 214 (87%)
South Asian (SA)	5408 (13%)	4243 (78%)***	2910	2241 (77%)***
Unknown/Other (U/O)	3420 (8%)	2351 (69%)***	1891	1726 (91%)***
Afro‐Caribbean (AC)	1792 (4%)	1391 (78%)***	973	784 (81%)***
Diabetes coding				
Prior	5867 (14%)	5523 (94%)***	3080	—
No prior	37 334 (86%)	26 404 (71%)	18 965	18 965 (100%)
Admission <24 h	14 181 (33%)	8258 (58%)***	6224	5524 (89%)***
Admission ≥24 h	29 020 (67%)	23 669 (82%)	15 821	13 441 (85%)
Repeat admission	12 537 (29%)	9882 (79%)***	—	—
Glucose^d^ mmol/L	—	6.4 (5.4‐8.0)	6.4 (5.4‐7.9)	6.2 (5.3‐7.4)***

^a^ % for categories within column.

^b^ *P* values for comparing % in each category; WE, reference category for ethnicity.

^c^ *P* values for comparison of glucose for column 4 vs those in 3 but not 4.

^d^ Median and quartiles otherwise n (%).

^e^ *P* values for comparison of age—column 2 vs those in 1 but not 2 and column 4 vs those in 3 but not 4.

*** *P* < .001.

**TABLE 2:** Ethnic differences in people admitted as an emergency with glucose measured on admission but no prior diagnosis of diabetes

	People with no prior diabetes coding and glucose available^a^	White European (WE)	South Asian (SA)	Unknown/Other (U/O)	Afro‐Caribbean (AC)	*P* values
n	18 965	14 214	2241	1726	784	
Age^b^, y	58 (38‐76)	62 (43‐78)	46 (32‐64)	43 (29‐61)	49 (33‐63)	c***
Age ≥90 y	968 (5%)	906 (6%)	31 (1%)	24 (1%)	7 (1%)	
Female, n (%)	9539 (50%)	7245 (51%)	1136 (51%)	761 (44%)	397 (51%)	d***
Male	9325 (49%)	6969 (49%)	1105 (49%)	864 (50%)	387 (49%)	
Not recorded	101 (1%)	0 (0%)	0 (0%)	101 (6%)	0 (0%)	
Glucose^b^ mmol/L	6.2 (5.3‐7.4)	6.2 (5.4‐7.5)	6.2 (5.3‐7.5)	5.9 (5.2‐7.2)	6.0 (5.2‐7.2)	e***
Ranges, n (%)						
<5.0	2672 (14%)	1868 (13%)	364 (16%)	291 (17%)	149 (19%)	
5.0‐5.5	3157 (17%)	2309 (16%)	375 (17%)	332 (19%)	141 (18%)	
5.6‐7.7	9091 (48%)	6970 (49%)	989 (44%)	778 (45%)	354 (45%)	
7.8‐11.0	3042 (16%)	2379 (17%)	338 (15%)	236 (14%)	89 (11%)	
>11.0	1003 (5%)	688 (5%)	175 (8%)	89 (5%)	51 (7%)	f***

^a^ Single/index if multiple admissions.

^b^ Median, IQ range.

*** *P* < .001 for ^c^WE vs SA, WE vs U/O, WE vs AC, SA vs U/O, U/O vs AC; ^d^WE vs U/O after excluding ‘not recorded’; ^e^WE vs U/O & AC, SA vs U/O & AC (WE & SA not significantly different nor U/O & AC); ^f^for proportion with glucose >11.0 mmol/L for WE vs SA, U/O vs SA.

Age bands of 15‐19 years, 20‐24 years, 25‐29 years up to 95‐99 years and 100‐104 years were used to construct Figure 3A which shows the proportion of patients in each age band by ethnicity. For Figure 3B,C, predicted glucose values obtained from a linear regression model of log glucose on age, sex and ethnicity (including interactions, Table 3) were plotted against age. Differences between the sexes and between ethnic groups were assessed by examining their interaction with age. No adjustment was made for multiple comparisons.

**TABLE 3:** Equations relating glucose in mmol/L to age in years for each sex for the different ethnic groups

	White European (WE)	South Asian (SA)	Unknown/Other (U/O)	Afro‐Caribbean (AC)
Males, n	6966	1104	863	387
Females, n	7244	1136	761	397
Males equation	log_10_ glucose = 0.00094 × age + 0.761	log_10_ glucose = 0.00177 × age + 0.743	log_10_ glucose = 0.00143 × age + 0.743	log_10_ glucose = 0.00105 × age + 0.758
*P* value	<.001^a^	<.001^b^	<.05^b^	<.05^a^
Females equation	log_10_ glucose = 0.00129 × age + 0.732	log_10_ glucose = 0.00230 × age + 0.694	log_10_ glucose = 0.00147 × age + 0.720	log_10_ glucose = 0.00229 × age + 0.686
*P* value	—	<.001^b^	—	<.01^b^

^a^ Comparison of age coefficient to that for females from the same ethnic group.

^b^ Comparison of age coefficient to that for White Europeans of the same sex.

Bonferroni‐corrected *t* tests were performed to investigate age and gender differences in admission plasma glucose in nonrepeat admissions within each ethnic group. Multilevel contingency tables associating frequencies of glucose, sex, and ethnicity (Figure 4A), and glucose, sex, and age group within the ethnic groups (Figure 4B‐E) were analysed with Pearson's chi‐square test for independence.

Power calculations were performed using sample sizes for people in ethnic groups in Table 2 with a significance level of .05/6 used to adjust for multiple comparisons. Due to unequal sample size, the power to detect a difference depended on whether the lower proportion related to the smaller or larger group.

## 3 RESULTS

### 3.1 People admitted as an emergency

Of the 113 097 admissions over 1 year, 38% (43 201) were emergency (Figure 1). Plasma glucose was measured on admission in 74% (31 927) (Table 1), with 75% from blood glucose meters, 18% blood gas machines and 7% laboratory analysers. Out of the 5867 admissions with a prior diabetes diagnosis, 94% (5523) had plasma glucose reported.

### 3.2 Availability of glucose

One third of all emergency admissions stayed in hospital for <24 hours and two thirds for >24 hours; 58% of emergency admissions with a duration <24 hours had glucose available and 82% of emergency admissions with duration >24 hours (Table 1). Admissions with plasma glucose reported (31 927) were 2 years older than the total cohort (43 201), median, IQ range for age, 62 (43‐77) vs 60 (41‐77) years, *P* < .001 (Table 1), with no differences in gender. Admissions without glucose measurements were 8 years younger at 54 (36‐73) years, *P* < .001.

A substantial number of emergency admissions, 12 537, were re‐admissions and glucose was measured in 79% (9882) of these. A total of 30 664 people were admitted with 76% (23 411) admitted once and 24% (7253) readmitted. Glucose was not measured on admission in 28% (8619) of these people who were younger at 53 (34‐71) years, than those with glucose available, 72% (22 045), aged 60 (41‐76) years, *P* < .001.

### 3.3 Timing of admission

Glucose was more likely to be measured on admission to Queen Elizabeth Hospital Birmingham at the weekend (Saturday/Sunday), 76% vs 73%, *P* < .001, or during the week between 6 pm and 6 am, 76% vs 71%, *P* < .001.

### 3.4 Multiple admissions

People readmitted (7253) were 9 years older than those admitted once (23 411), *P* < .001 with a higher proportion of women, 52% vs 49%, and White Europeans, 79% vs 73%, and fewer from Unknown/Other ethnic groups, 5% vs 10%, all at *P* < .001. Of these re‐admissions, 63% (4569) were on two occasions, 20% (1472) three, 9% (630) four and 8% (582) on five or more occasions. People readmitted were more likely to be coded for diabetes, 17% (1266) vs 9% (2045), *P* < .001. The prevalence of prior diabetes increased with the number of admissions, that is 15% (689) for two, 18% (272) for three, 23% (146) for four and 27% (159) for five or more admissions, Kendall's tau‐*b* = 0.09, *P* < .001.

### 3.5 Glucose and glycaemic status on admission

Glucose was 6.4 (5.4‐8.0) mmol/L in 74% (31 927) of admissions with glucose available (Table 1); 8.8 (6.6‐12.5) mmol/L in 17% (5523) of these admissions with prior diabetes coding and 6.2 (5.3‐7.4) mmol/L in 83% (26 404) with no diabetes coding. In admissions without prior diabetes coding, 31% (8059) were ≤5.5 mmol/L, 48% (12 704) 5.6‐7.7 mmol/L, 16% (4283) 7.8‐11.0 mmol/L, that is ‘at risk’ range and 5% (1358) ≥11.1 mmol/L ‘diabetes’ range.

**FIGURE 2:**
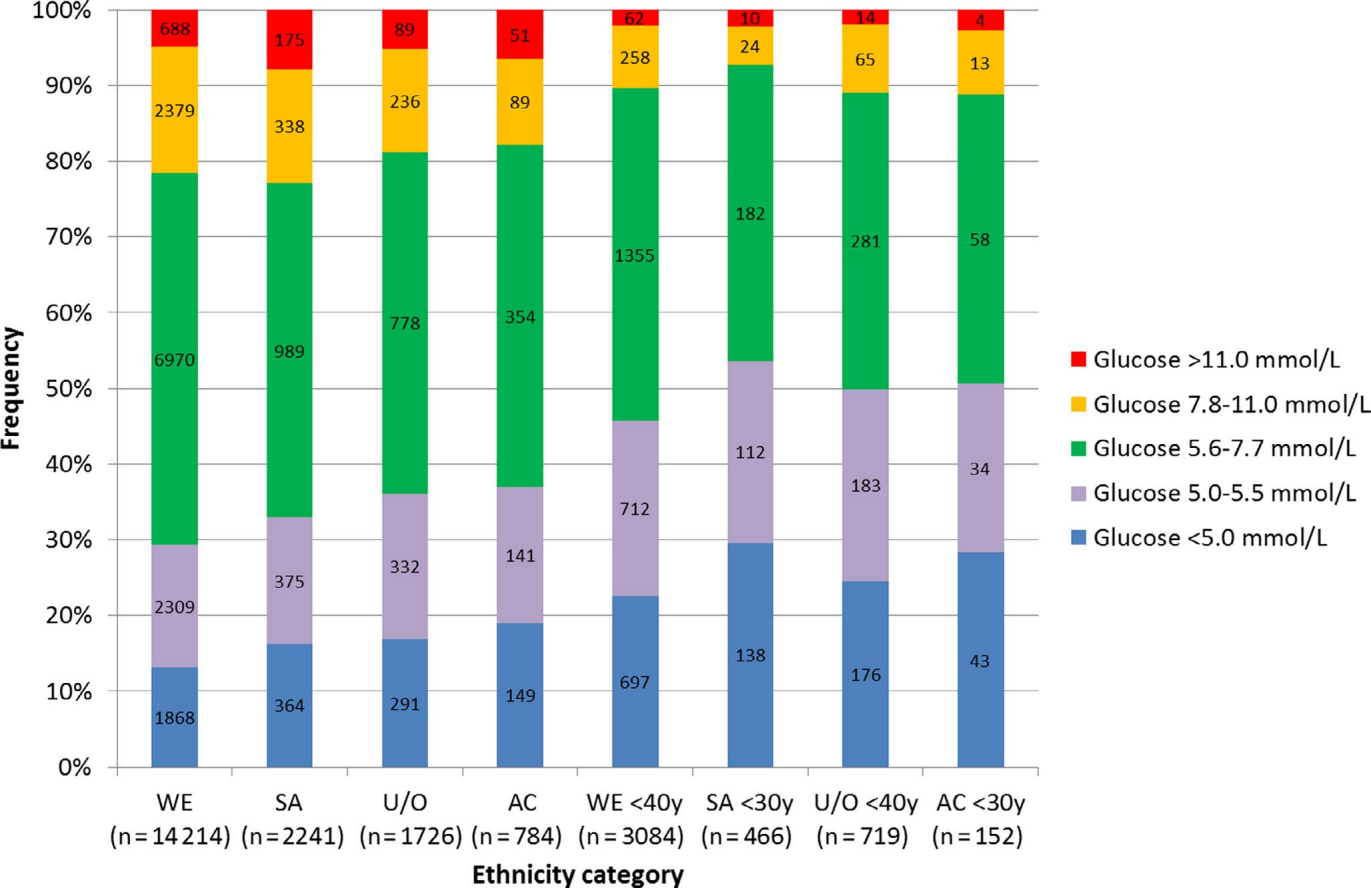
Glucose ranges for people admitted as an emergency without prior diabetes diagnosis by ethnicity and proposed age limit for follow‐up. AC, Afro‐Caribbean; SA, South Asian; U/O, Unknown/Other; WE, White European

Over 20% of the people admitted as an emergency had hyperglycaemia; 5% had glucose in the ‘diabetes’ range and 16% in the ‘at risk’ range, Table 2 and Figure 2 with a higher proportion of South Asians (8%) than White Europeans (5%) in the ‘diabetes’ range, *P* < .001. The proportion of White Europeans (17%) and South Asians (15%) in the ‘at risk’ range was higher than for Afro‐Caribbeans, (11%) *P* < .001 and *P* = .010. Some guidance specifies age limits below which people should not be tested for undiagnosed diabetes.^15^ For South Asians aged <30 years, glucose was in the ‘diabetes’ and ‘at risk’ ranges for 2.1% and 5.2%, respectively, for Afro‐Caribbeans aged <30 years 2.6% and 8.6%, for Unknown/Others aged <40 years 1.9% and 9.0%, and for White Europeans aged <40 years 2.0% and 8.4%. When people below these age limits were excluded, the prevalences were 6.3% and 18.4% compared with 5.3% and 16.0% when all ages were included.

### 3.6 Ethnicity of people admitted

**FIGURE 3:**
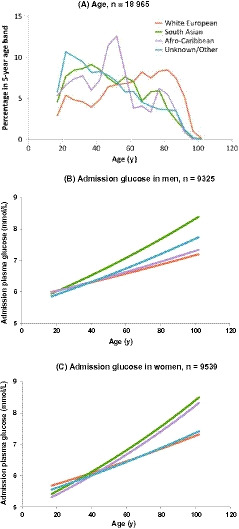
Age distribution and predicted admission glucose of people admitted to hospital as an emergency without prior diabetes diagnosis by ethnicity. Purple: Afro‐Caribbean; green: South Asian; blue: Unknown/Other ethnic groups; orange: White European

Three quarters of people admitted without prior diabetes coding with glucose available were White European, median age 62 years (Table 2). They were older than 12% of South Asians (46 years), 9% when ethnicity Unknown/Other, (43 years) and 4% Afro‐Caribbean (49 years), *P* < .001. However, the age distributions of the ethnic groups were markedly different (Figure 3A). Overall, 5% (968) of people were aged 90 years old or older with 6% White European and 1% from the other ethnic groups (Table 2). Proportionally glucose was available for more South Asian and Afro‐Caribbean admissions, 78% vs 73% for White European and 69% for Unknown/Others (Table 1).

### 3.7 Admission glucose by age, gender and ethnicity

Glucose was higher as the age of the people admitted increased (Figure 3B,C), and varied by ethnicity. South Asian men aged >21 years and women aged >37 years had higher glucose than the White Europeans. In terms of overall plasma glucose levels, White Europeans and South Asians had slightly higher median glucose on admission at 6.2 mmol/L than Afro‐Caribbeans, 6.0 mmol/L, and Unknown/Others 5.9 mmmol/L, *P* < .001. There were significant differences in glucose over the age distribution depending on people's gender and ethnicity (Table 3). Increases in glucose with age were greater for South Asian men and women than for White Europeans of the same gender; also for men whose ethnicity was Unknown/Other and Afro‐Caribbean women (Table 3).

**FIGURE 4:**
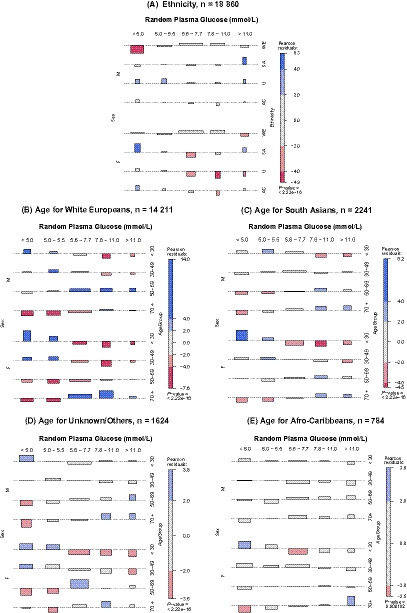
Glucose levels in people admitted to hospital as an emergency without prior diabetes diagnosis by gender, ethnicity and age. M, male; F, female. Blue—frequency observed significantly more than expected with positivity increasing with depth of colour; red—less frequently; grey—nonsignificant residuals. Boxes—areas proportional to difference in observed and expected frequencies; dashed grey baseline is expected count; above baseline greater than expected frequencies; below fewer than expected frequencies. Height is proportional to contribution of Pearson's residuals; width is proportional to square root of expected counts

When relating ethnicity to glucose ranges, South Asian men were more prevalent than expected in the 'diabetes' range, >11.0 mmol/L, and South Asian women in the lowest range, <5.0 mmol/L (Figure 4A). A similar analysis with age showed that South Asian women aged <30 years were most prevalent in the lowest glucose range, with South Asian men and women aged >70 years more prevalent in the 'diabetes' and 'at risk' ranges (Figure 4C). White European men aged 50‐69 years old were prevalent in the 'diabetes', 'at risk' and 'prediabetes' ranges (Figure 4B).

### 3.8 Diabetes diagnosed during hospital admission

Diabetes was diagnosed following routine protocol during the index admission in people without a prior diagnosis before admission (and with glucose measured) in 10% (1849/18 965), that is in 8% (1184/14 214) of White Europeans, 10% (168/1726) Unknown/Other ethnic groups, 14% (108/784) Afro‐Caribbeans and 17% (389/2241) South Asians. Of the 1849 newly diagnosed, 575 were in the 'diabetes' range (31%) and 495 were in the 'at risk' range (27%).

## 4 DISCUSSION

The prevalence of diabetes recorded in people in hospital in Birmingham is nearly double that of the community at 22% vs 12%.^16^ Early diagnosis of diabetes is important as people can be advised to alter their diet, exercise regimen and lifestyle, or blood glucose lowering treatment can be introduced when necessary.

Hyperglycaemia on admission to hospital is defined as 'at risk' of diabetes, that is 7.9‐11 mmol/L or in the 'diabetes' range, that is >11 mmol/L. Immediate action is required on the ward when people present with very high glucose, for example 25/30 mmol/L^15^ to prevent/diagnose life‐threatening conditions such as diabetic ketoacidosis.

Undiagnosed diabetes may be the cause of hyperglycaemia on admission but its diagnosis should be confirmed by additional testing with HbA1c. In this audit, 5% of White Europeans and 8% of South Asians and Afro‐Caribbeans had glucose in the 'diabetes' range but there is little evidence on how many cases of diabetes would be confirmed on HbA1c testing. People in this study below the age limits specified in some guidance on additional testing had glucose in the abnormal ranges.

National protocols for identifying undiagnosed diabetes in admissions are mainly based on expert opinion and do not address the entire process from flexi‐testing in a hospital laboratory to follow‐up by GPs. Medico‐legal implications can arise when abnormal glucose is not acted on during admission as people may present some years later with diabetes complications if not diagnosed during or after their hospital stay (Personal communication from Dr Sandip Ghosh and Professor Graham Roberts). Some preliminary data on those diagnosed with diabetes on admission using routine procedures are presented here. But, it requires more attention by the research team as it could reflect coding practice and is included in an ongoing project.

On emergency admission, 94% of those admitted to this hospital with a previous diabetes diagnosis had glucose recorded, but the figure for those not previously diagnosed was 74%. How this performance compares with other UK hospitals could be assessed by national inpatient audit programs. This figure may be related to the length of stay in hospital. One third of all emergency admissions were for <24 hours with 58% of these having glucose measured compared to 82% with a duration of >24 hours (Table 1). Those without glucose available were younger and more likely to be White European. The time/day of admission did not markedly influence the availability with only small differences observed possibly reflecting the hospital organization.

The number of people in the 'at risk' range was much higher than those in the 'diabetes' range involving 16% of people admitted with glucose measured. However, this audit is limited by the length of time the various ethnic groups have resided in the West Midlands.^17^ As South Asians and Afro‐Caribbeans present with diabetes at a younger age than White Europeans, it is vital to consider the age cut‐offs for further testing quoted in some cases as 30 years for the former groups and 40 years for latter.^15^ In routine practice, we have identified South Asian males presenting in their 20s with very high glucose and diabetic ketoacidosis—the reason why this audit was generated. As there were fewer Afro‐Caribbeans in the audit, the power to detect differences between proportions of 5% and 8% in this group was lower at 54%‐78% than for the other ethnic groups, 87%‐100%.

The data presented here suggest that there should be no lower limit for follow‐up testing in adults (Figure 2). This is important as diagnosis impacts on both disease progression and the risk of developing complications which are apparent several years before diagnosis. This evidence will help guideline writers to assess the workload and cost of implementing procedures for diagnosing diabetes in emergency admissions. The Joint British Diabetes Societies (JBDS) guidance on diabetes at the front door issued in February 2020^15^ recommends further testing with HbA1c if glucose >7.8 mmol/L in people aged 40 years or older or 30 years depending on ethnicity.

Upgrading electronic patient record systems to identify people with glucose in ‘diabetes’ range and to prioritize further HbA1c testing could help reduce readmission rates. As raised glucose on admission was a particular problem in re‐admissions, only their first admission was included in subsequent analyses. The prevalence of admission glucose in the 'diabetes' range was 27% for those admitted on five or more occasions.

Medical history/current records should be accessed when following up raised admission glucose as conditions requiring emergency hospitalization can cause anaemia which may affect the accuracy of HbA1c.^18^, ^19^ Ethnic differences in its relationship with glucose may be linked to red blood cell morphology.^20^, ^21^ In addition, variation in people with normal haematological profiles can account for differences of up to 5 mmol/mol (0.5%). Some countries are questioning whether different HbA1c cut‐offs are necessary for diagnosis of diabetes. When inaccuracy is suspected, fructosamine can confirm abnormal glucose levels but the test is not recommended for diagnosis.

HbA1c is requested on admission of people without diagnosed diabetes now in some hospitals in Europe, America and Australia but published data on its efficacy is minimal.^6^, ^22^ When diagnosis using HbA1c was compared with OGTT if fasting glucose raised in general practice, correlation on diabetes diagnosis reached 95% when HbA1c >57 mmol/mol (7.5%).^23^ A recent study of Australian adults aged ≥60 years reports a low diagnosis rate for diabetes in emergency hospital admissions due to people going into hospital undiagnosed and remaining undiagnosed during admission or with HbA1c results not necessarily communicated to family doctors on discharge.^24^

## 5 CONCLUSIONS

A significant number of people admitted as an emergency but not previously diagnosed with diabetes had hyperglycaemia within the ‘diabetes’ (5%) and ‘at‐risk’ ranges (16%) (Figure 2). South Asians were admitted at a younger age than White Caucasians with their admission glucose higher and South Asian men particularly affected (Figure 4A). This audit highlighted various issues regarding the availability of glucose on admission (75%), readmission rate as hyperglycaemia increased with the number of admissions, whether age limits should be employed for additional HbA1c testing to confirm diagnosis as people below limits specified had admission glucose in the abnormal ranges, and the effect of the length of time the various ethnic groups have resided in the West Midlands. Further investigation into the efficacy, procedures and cost of diagnosis in emergency admissions is required—this will involve reflex HbA1c testing and algorithms linking hospital and primary care. Liaison between public health, diabetes organizations and researchers is required to address these issues.

## CONFLICT OF INTEREST

There are no conflicts of interest for the authors. The study sponsor, University Hospitals Birmingham NHS Foundation Trust, was not involved in the design of the study; the collection, analysis and interpretation of data; writing the report; or the decision to submit the report for publication.

## AUTHOR CONTRIBUTIONS

All authors qualify for authorship based on the International Committee of Medical Journal Editors criteria. All authors take full responsibility for content of manuscript. SG designed and organized the clinical audit and reviewed data and manuscript. SEM was responsible for data analysis and writing the manuscript. I.A‐P. created the database. PGN and JAW analysed data and edited the manuscript. IMS and GVG reviewed the data analyses and the manuscript. RS contributed to data analysis and interpretation, and writing the manuscript. JW reviewed clinical aspects and also the manuscript. SDL contributed to data analysis and interpretation, and reviewed the manuscript. GAR and WH contributed to the overall audit process and reviewed clinical aspects of the paper. SG is the guarantor of this work.

## Data Availability

The data sets generated during and/or analysed during the study are not publicly available. The data set contains clinical data which cannot be shared publicly due to UK data protection legislation.
